# Positive additive interaction effects of age, sex, obesity, and metabolic syndrome on left ventricular dysfunction

**DOI:** 10.1111/1753-0407.13478

**Published:** 2023-09-24

**Authors:** Dan Zhou, Zhongwen Ye, Zhiqiang Nie, Chaolei Chen, Songyuan Luo, Mengqi Yan, Jiabin Wang, Yingqing Feng

**Affiliations:** ^1^ Department of Cardiology Guangdong Provincial Key Laboratory of Coronary Heart Disease Prevention, Guangdong Cardiovascular Institute, Guangdong Provincial People’s Hospital (Guangdong Academy of Medical Sciences), Southern Medical University Guangzhou China; ^2^ Department of Internal Medicine Shenzhen People's Hospital (The Second Clinical Medical College, Jinan University; the First Affiliated Hospital, Southern University of Science and Technology) Shenzhen China; ^3^ Zhuhai Center for Disease Control and Prevention Zhuhai China; ^4^ Global Health Research Center, Guangdong Provincial People's Hospital, Guangdong Academy of Medical Sciences Southern Medical University Guangzhou China

**Keywords:** global longitudinal strain, interaction effect, left ventricular dysfunction, metabolic syndrome

## Abstract

**Objective:**

This study aims to explore the association between metabolic syndrome (MetS) and left ventricular diastolic dysfunction (LVDD) and systolic dysfunction (LVSD), defined by impaired global longitudinal strain (GLS), and assess additive and multiplicative interactions among age, sex, obesity, and MetS regarding LVDD and LVSD.

**Methods:**

We prospectively recruited 5503 participants from the China PEACE (Patient‐Centered Evaluative Assessment of Cardiac Events) Million Persons Project with complete echocardiography exam. Multivariable logistic models were used to calculate adjusted odds ratios to evaluate both additive and multiplicative interactions.

**Results:**

The mean age was 56.59 years; 59.4% were women, 46.7% had MetS, 26.6% had left ventricular hypertrophy, 46.3% had LVDD, and 12.50% had impaired GLS. Compared to the non‐MetS, the odds ratio (OR) of LVDD and impaired GLS in MetS were 1.40 (1.20–1.64) and 1.26 (1.03–1.54), respectively. For LVDD, relative excess risk due to additive interaction (RERI) for women and MetS, elderly and MetS, obesity and MetS were 0.76 (0.02–1.50), 35.65 (17.51–53.79), and 2.14 (0.66–3.62), respectively, thus suggesting additive interactions. For impaired GLS, RERI for obesity and MetS was 3.37 (0.50–6.24), thus suggesting additive interactions.

**Conclusions:**

The MetS is independently associated with LVDD and impaired GLS. From the public health implications, prevention of MetS in women, elderly, and obese individuals might result in a greater reduction of LVDD and LVSD risk in cardiovascular high‐risk population.

## Graphical abstract

**Highlights**

- Left ventricular dysfunction such as left ventricular diastolic dysfunction (LVDD) and impaired global longitudinal strain (GLS) were important causes of cardiac events in cardiovascular high‐risk population.
- Metabolic disorders play a key role in the progression of left ventricular dysfunction, therefore, the prevention of metabolic syndrome (MetS) in women, elderly, and obese individuals might reduce the burden of LVDD and left ventricular systolic dysfunction.
- The study first analyzed LVDD and GLS by echocardiography in a large community population, providing new insights for community target organ damage screening in MetS.

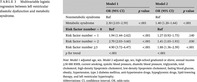

## 1 INTRODUCTION

Metabolic syndrome (MetS) is a complex syndrome that includes obesity, hypertension, diabetes, insulin resistance, and dyslipidemia that increases cardiovascular diseases. There is a growing body of evidence that MetS causes myocardial structural changes and contractile dysfunction, including left ventricular diastolic dysfunction (LVDD) and systolic dysfunction (LVSD), defined by impaired global longitudinal strain (GLS),^1^ and increases cardiovascular disease.^2^

In Hispanics/Latinos, MetS has been associated with increased ratio of peak mitral inflow velocity and early diastolic mitral annulus velocity (E/e' ratio), decreased e', and decreased left ventricular longitudinal strain in apical two‐ and four‐chamber views.^3^ The Brazilian Longitudinal Study of Adult Health (ELSA‐Brasil study)^4^ found MetS was closely associated with impaired GLS evaluated by average apical two‐ and four‐chamber views in participants without overt heart disease. In China, the prevalence of MetS was 32.3% in a nationwide cross‐sectional study from 2012 to 2015 by revised Adult Treatment Panel III definition.^5^ There is limited information on the association between MetS and LVSD and LVDD, evaluated by GLS and e', E/e' ratio and left atrial volume index (LAVI)^6^ in cardiovascular high‐risk population. Previous study reported women had worse LV diastolic function^7^ and obesity was associated with decreased GLS and diastolic dysfunction such as lower e' and higher E/e' ratio.^8^

Given the major MetS epidemic and related cardiovascular disease and paucity of studies examining the impact of MetS on LVDD and impaired GLS, our aim was to explore the association between MetS and LVDD and impaired GLS. In particular, little is known about interaction effects of MetS and age, sex, and obesity on LVDD and LVSD, which is the focus of our study. The presence of a positive additive interaction may contribute to the preventive strategies and public health policies, even with negative or no multiplicative interactions.^9^ The purpose of this study was to assess additive and multiplicative interactions among combinations of age, sex, obesity, and MetS, using LVDD and LVSD as the outcome.

## 2 METHODS

### 2.1 Study participants

**FIGURE 1:**
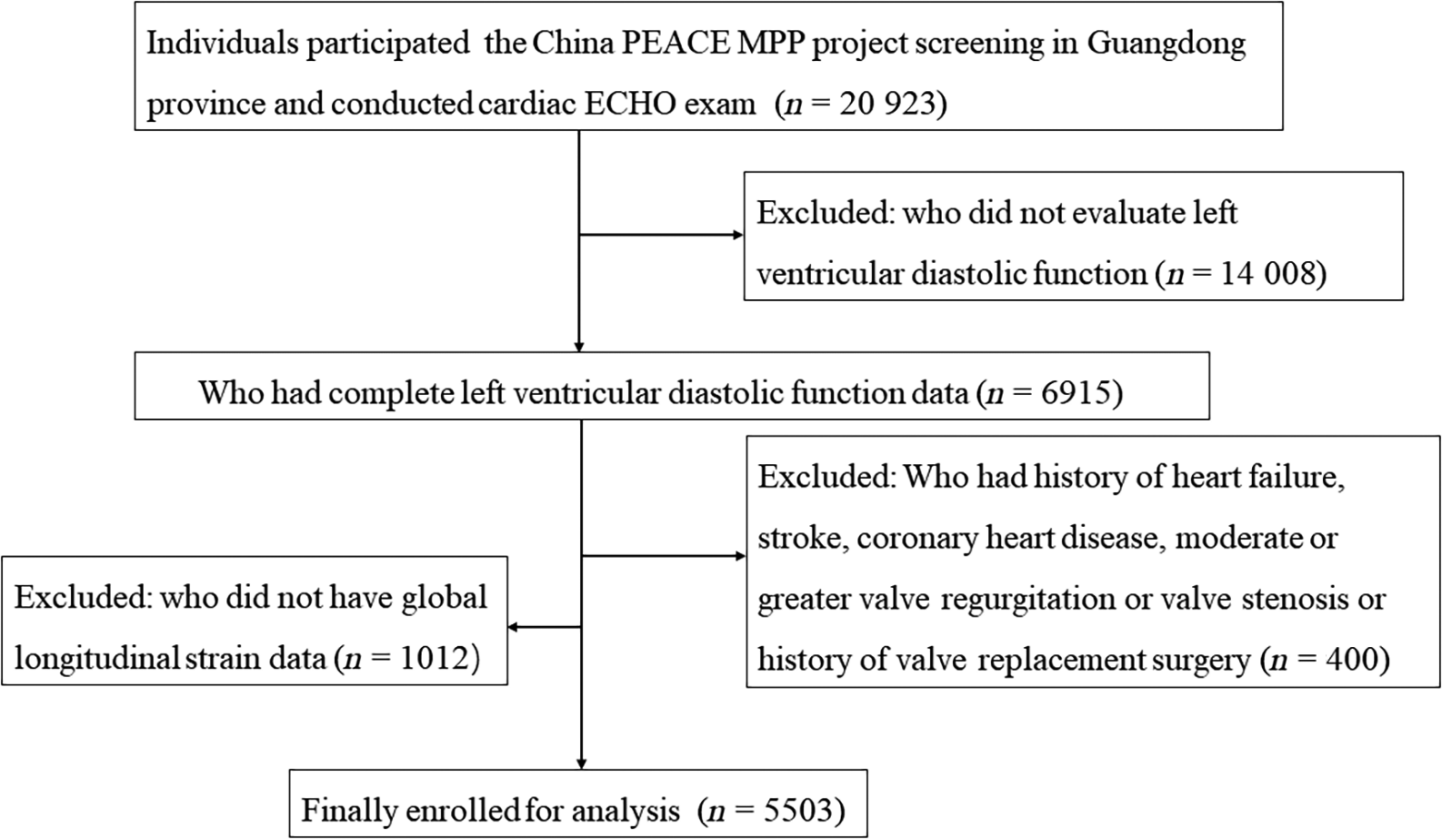
Study flow chart. ECHO, echocardiogram; MPP, Million Persons Project; PEACE, Patient‐Centered Evaluative Assessment of Cardiac Events.

We prospectively recruited individuals aged 35 to 75 years from the China PEACE (Patient‐Centered Evaluative Assessment of Cardiac Events) Million Persons Project, which was sponsored by the Chinese government, as previously published.^10^ The inclusion criteria were (1) the subjects at high risk of cardiovascular disease as the protocol described^10^ and (2) willing to complete a cardiac ultrasound examination. Between January 1, 2016 and December 31, 2019, we prospectively conducted cardiac echo examination in 20 923 participants in eight sites across Guangdong Province. We excluded participants who did not evaluate LV diastolic function (*n* = 14 008), and who had history of heart failure, stroke, coronary heart disease, moderate or greater value regurgitation or valve stenosis, or history of valve replacement surgery (*n* = 400), and who did not have GLS data (*n* = 1012). Finally, we enrolled 5503 participants for analysis (Figure 1). The research protocol was approved by the Central Ethics Committee at the China National Center for Cardiovascular Disease and the Ethics Committee of Guangdong Provincial People's Hospital [No. GDREC2016438H (R2)]. Additionally, all participants provided written informed consent.

### 2.2 Covariates

We used the Nationwide Survey in China Primary Health Care Organization questionnaire to collect the sociodemographic information. The community health care staffs were trained to collect the information with electronic questionnaire system, including sex, age, married or not, education status, current smoking, current drinking, economic status, as well as comorbidities (hypertension, type 2 diabetes mellitus) and current medications such as hypoglycemic, antihypertensive drugs and lipid‐lowering therapy.

The body surface area (BSA) was determined as follows: BSA (m^2^) = (weight [kg]^0.425^ * height [cm]^0.725^) * 0.007184.^11^ Body mass index (BMI) was calculated as weight(kg)/[height(m)].^2^ Obesity was identified with BMI ≥28 kg/m^2^.^12^ Waist circumference was measured at the level of the midpoint between the top of the iliac crest and the lower margin of the last palpable rib in the midaxillary line. Blood pressure (BP) was measured twice within 1 min interval by trained community healthcare staffs using a validated automated BP monitor (Omron HEM‐7430, Omron Corporation, Kyoto, Japan) and the average value was used. Hypertension^13^ was defined as those who self‐reported history of hypertension or systolic blood pressure (SBP) ≥140 mm Hg or diastolic blood pressure (DBP) ≥90 mm Hg or the use of antihypertensive medicine within 2 weeks. Fingertip whole blood samples were used to measuring fasting blood glucose (FBG) (BeneCheck BK6–20 M Multi‐Monitoring System, Suzhou Pu Chun Tang Biotechnology, China) and common lipid profiles (CardioChek PA Analyzer; Polymer Technology Systems, Indianapolis, Indiana, USA). Self‐reported type 2 diabetes mellitus or using hypoglycemic drugs or FBG ≥ 7.0 mmol/L was defined as type 2 diabetes mellitus.^14^ Participants were considered in a fasting state if their last meals were taken more than 8 h before. Total cholesterol, triglyceride (TG) and high‐density lipoprotein cholesterol (HDL‐C) were measured. Low‐density lipoprotein cholesterol (LDL‐C) was calculated with the Friedewald equation after excluding participants with TG greater than 400 mg/dL (to convert TG to mmol/L, multiply by 0.0113).^11^

### 2.3 Metabolic syndrome

MetS was defined as presence of any three of following five elements.^15^ Abdominal obesity was defined as waist circumference (WC) ≥ 90 cm (male) or ≥ 85 (female) in Chinese. Elevated TG was defined as TG ≥ 150 mg/dL. Reduced HDL‐C was defined as HDL‐C < 40 mg/dL (male) or HDL‐C < 50 mg/dL (female). Elevated BP was defined as SBP ≥ 130 and/or DBP ≥ 85 mm Hg or using antihypertensive drugs. Elevated FBG was defined as FBG ≥ 100 mg/dL or using hypoglycemic drugs.

### 2.4 Echocardiographic examination

Guangdong provincial people's hospital sonographer and local ultrasound physicians conduct echocardiography examination in accordance with standards set down by the National Health and Family Planning Commission, China, as previous protocol reported.^10^ All echocardiogram imaging was saved as DICOME and was reviewed by Guangdong provincial people's hospital experts. Stored imaging should include M‐mode and 2‐D measurements, Doppler flow parameters and tissue Doppler imaging. Echocardiographic analysis was performed by three experienced sonographers from Guangdong provincial people's hospital using standard methodologies with an offline system (GE Echo PAC201).

Left ventricular end‐diastolic diameter (LVEDD), Interventricular septum thickness (IVS), Left ventricular posterior wall thickness (LVPW), and left ventricular ejection fraction were acquired at the level of the mitral valve leaflets from the parasternal long‐axis view. Left ventricular mass (LVM) was defined as LVM = 0.8 * 1.04 * [(IVS + LVEDD + LVPW)^3^ − LVEDD^3^] + 0.6 g.^16^ Relative wall thickness (RWT) was defined as RWT = (LVPW * 2)/LVEDD. We calculated LVM index (LVMI) by index BSA. LVH was defined as LVMI > 115 g/m^2^ (male) or > 95 g/ m^2^ (female). From the four‐chamber view, we acquire peak E wave and A wave of mitral inflow velocity. Peak early diastolic tissue velocity (e') was acquired at the interventricular septum (septal e' velocity) and lateral wall (lateral e' velocity) of the mitral annulus. LAV was acquired from four‐chamber and two‐chamber and the average value was used. LAVI was calculated by index BSA. E/A ratio, septal e' velocity, lateral e' velocity, average E/e' ratio and LAVI were used to evaluate LVDD.^17^ Among septal e' < 8 cm/s, lateral e' < 10 cm/s and LAVI ≥34 mL/m^2^, the presence of two abnormal measurements was defined as LVDD. The LVDD grade was defined according to the mitral E/A and average E/e' ratio. In this study, the proportion of grade II or III diastolic dysfunction was relatively low. Thus, we divided the participants into LVDD and non‐LVDD for analysis. Average GLS was measured from the apical four‐chamber, two‐chamber, and three‐chamber views^18^ using two‐dimensional speckle tracking technology. We use the absolute value of GLS in the text. Impaired GLS was defined as GLS < 16% as previous recommended.^6^

### 2.5 Statistical analysis

Characteristics of the study participants were described by MetS and MetS elements numbers. Continuous variables were described as mean ± standard deviation and categorical variables were described as number and percentage after verifying the normal distribution using Kolmogorov–Smirnov test. Study participants' characteristics were compared using chi‐square tests and one‐way analysis of variance tests. Linear regression was used to evaluate the linear relationship between the continuous variables and the number of different risk elements. A Cochran–Armitage trend test was used to analyze the relationship between dichotomous variables' positive rate and different MetS elements number. We used logistic models to estimate odds ratios (ORs) and 95% confidence intervals (CIs) of LVDD and impaired GLS for MetS compared to non‐MetS and ≥3 MetS elements, 2 MetS elements, 1 MetS element compared to 0 MetS elements. Model 1 was adjusted for age and sex; Model 2 with additional adjustment for other covariables. Collinearity test and variance inflation factor value was showed in Table S1.

The stratification analyses were carried out based on sex (men vs women), age (elderly ≥60 years vs < 60 years), obesity (yes vs no). Logistic regression model was used to estimate the joint association of sex, age, obesity, and MetS on the risk of LVDD and impaired GLS. We measure interaction on both additive and multiplicative scales. The coefficients of product term (sex * MetS, age * MetS, obesity * MetS) in multivariable logistic regression could reflect the multiplicative scales.^19^ Age was considered as a two‐category variable with ≥60 years vs < 60 years. When the interaction ratio >1, a positive multiplicative interaction is present.

Additive interaction was measured by three indicators including (a) relative excess risk due to interaction (RERI), (b) attributable proportion due to interaction (AP), and (c) synergy index (S).^20^ There was a synergistic additive interaction when RERI > 0 and AP >0, or S > 1. There was an antagonistic interaction when RERI<0 and AP <0, or S < 1.

According to the guideline recommendation, glycemic, lipidemic, and BP targets were <7 mmol/L, LDL‐C < 2.6 mmol/L^21^ and < 140/90 mm Hg, respectively. We further grouped MetS participants into MetS with all controlled glycemic, lipidemic, and BP and MetS with uncontrolled glycemic, lipidemic, or BP. We supplied the sensitivity analysis with elevated FBG in a MetS definition that was defined only by using hypoglycemic drugs. All analyses were conducted with SPSS statistical software version 27 and R statistical software version 4.2.2 (R Project for Statistical Computing). *p* < .05 was considered significant.

## 3 RESULTS

### 3.1 Characteristics of study population

Among the 5503 participants, the median age was 56.59 years, 46.7% had MetS, 59.4% were women, 59.6% had hypertension, 19.4% had type 2 diabetes mellitus, 17.1% had obesity, 28.10% used antihypertensive drugs, 8% used hypoglycemic drugs, 4.5% had lipid‐lowering therapy (Table 1), 26.6% had LVH, 46.3% had LVDD, and 12.50% had impaired GLS (Table 1).

**TABLE 1:** Baseline characteristics of participants.

Variables	Total *N* = 5503	Nonmetabolic syndrome *N* = 2935	Metabolic syndrome *N* = 2568	*p* value
Age (years)	56.59 ± 9.74	55.54 ± 9.90	57.80 ± 9.41	<.001
Women, *n* (%)	3270 (59.4%)	1829 (62.3%)	1441 (56.1%)	<.001
High school graduated or above, *n* (%)	1351 (24.6%)	814 (27.7%)	537 (20.9%)	<.001
Annual income ≥ 50 000 RMB, *n* (%)	2666 (48.4%)	1423 (48.5%)	1243 (48.4%)	.487
Married, *n* (%)	5056 (91.9%)	2702 (92.1%)	2354 (91.7%)	.621
Current smoking, *n* (%)	1104 (20.10%)	543 (18.5%)	561 (21.8%)	.001
Current drinking, *n* (%)	298 (5.4%)	136 (4.6%)	162 (6.3%)	.004
Systolic blood pressure, mm Hg	142.86 ± 23.44	135.71 ± 23.82	151.03 ± 20.10	<.001
Diastolic blood pressure, mm Hg	82.64 ± 13.31	79.67 ± 13.30	86.04 ± 12.48	<.001
Heart rate, beat/min	80.37 ± 11.43	79.48 ± 11.20	81.39 ± 11.62	<.001
Body mass index, kg/m^2^	24.97 ± 3.41	23.59 ± 2.89	26.54 ± 3.27	<.001
Waist circumference, cm	86.03 ± 9.56	81.40 ± 8.06	91.33 ± 8.31	<.001
Total cholesterol, mmol/L	5.63 ± 1.44	5.69 ± 1.38	5.57 ± 1.51	.003
Triglyceride, mmol/L	1.90 ± 1.12	1.41 ± 0.69	2.47 ± 1.25	<.001
Low‐density lipoprotein cholesterol, mmol/L	3.30 ± 1.22	3.38 ± 1.20	3.20 ± 1.24	<.001
High‐density lipoprotein cholesterol, mmol/L	1.55 ± 0.48	1.72 ± 0.46	1.35 ± 0.42	<.001
Fasting blood glucose, mmol/L	6.11 ± 1.86	5.55 ± 1.33	6.74 ± 2.15	<.001
Medical therapy				
Antihypertensive drugs, *n* (%)	1547 (28.10%)	530 (18.1%)	1017 (39.6%)	<.001
Hypoglycemic drugs, *n* (%)	438 (8.00%)	82 (2.8%)	356 (13.9%)	<.001
Lipid‐lowering therapy, *n* (%)	250 (4.5%)	68 (2.30%)	182 (7.1%)	<.001
Complications				
Hypertension, *n* (%)	3281 (59.6%)	1308 (44.6%)	1973 (76.8%)	<.001
Type 2 diabetes mellitus, *n* (%)	1070 (19.40%)	235 (6.6%)	835 (32.5%)	<.001
Obesity, *n* (%)	943 (17.1%)	193 (6.6%)	750 (29.2%)	<.001
Echocardiographic parameters				
Left ventricular mass index, g/m^2^	91.01 ± 18.91	88.19 ± 18.25	94.24 ± 19.14	<.001
Left ventricular hypertrophy, *n* (%)	1463 (26.6%)	552 (18.8%)	911 (35.5%)	<.001
Left ventricular ejection fraction (%)	68.93 ± 5.42	68.90 ± 5.40	68.97 ± 5.45	.631
E, m/s	0.75 ± 0.18	0.78 ± 0.18	0.72 ± 0.18	<.001
E/A ratio	0.99 ± 0.35	1.07 ± 0.38	0.90 ± 0.30	<.001
Septal‐e', cm/s	7.51 ± 2.25	8.12 ± 2.34	6.81 ± 1.91	<.001
Lateral‐e', cm/s	10.01 ± 2.92	10.73 ± 3.00	9.18 ± 2.58	<.001
Septal‐E/e' ratio	10.63 ± 3.17	10.12 ± 2.94	11.22 ± 3.31	<.001
Lateral‐E/e' ratio	8.02 ± 2.59	7.68 ± 2.37	8.41 ± 2.77	<.001
Average‐E/e' ratio	9.33 ± 2.63	8.91 ± 2.45	9.82 ± 2.75	<.001
Left atrial volume index, mL/m^2^	24.88 ± 7.53	24.70 ± 7.55	25.08 ± 7.49	.063
Left ventricular diastolic dysfunction, *n* (%)	2549 (46.3%)	1067 (36.4%)	1482 (57.7%)	<.001
Global longitudinal strain, %	19.62 ± 3.70	20.26 ± 3.56	18.89 ± 3.73	<.001
Global longitudinal strain <16%, *n* (%)	688 (12.50%)	235 (8.00%)	453 (17.6%)	<.001

*Note*: Data are shown as median (Q1‐Q3) for continuous variables and number (percentage) for categorical variables.

### 3.2 Left ventricular structure and function parameters and MetS

Compared to the non‐MetS group, MetS individuals were elderly, men, lower educated, and more smokers and drinkers (Table 1). In Table 1, MetS individuals had increased LVMI, decreased E/A ratio, septal‐e’ and lateral‐e' velocity, slightly increased E/e' ratio and LAVI, and decreased GLS. MetS participants had remarkably higher proportion of LVH (35.5% vs 18.8%), LVDD (57.7% vs 36.4%), and impaired GLS (17.6% vs 8%) than non‐MetS participants.

Table 2 showed the left ventricular function parameters among four‐level categorical MetS elements groups. The decreasing E/A ratio, septal‐e', lateral‐e' velocity, and GLS were found with increasing MetS elements. Consistently, the increasing proportion of LVH, LVDD, and impaired GLS was found with increasing MetS elements (*p* for trend <.05).

**TABLE 2:** Baseline echocardiographic parameters between different number of metabolic risk factors.

Variables	Number of metabolic risk factors				*p* value	*p* for trend
	0 (*n* = 455)	1 (*n* = 1002)	2 (*n* = 1478)	3 to 5 (*n* = 2568)		
Left ventricular mass index, g/m^2^	82.02 ± 15.44	87.72 ± 17.60	90.40 ± 19.02	94.24 ± 19.14	<.001	<.001
Left ventricular hypertrophy, *n* (%)	39 (8.6%)	161 (16.1%)	352 (23.8%)	911 (35.5%)	<.001	<.001
Left ventricular ejection fraction (%)	69.32 ± 5.05	69.14 ± 5.41	68.60 ± 5.49	68.97 ± 5.45	.025	.893
E, m/s	0.82 ± 0.17	0.78 ± 0.18	0.76 ± 0.18	0.72 ± 0.18	<.001	<.001
E/A ratio	1.28 ± 0.41	1.10 ± 0.36	1.00 ± 0.35	0.90 ± 0.30	<.001	<.001
Septal‐e', cm/s	9.51 ± 2.35	8.29 ± 2.32	7.57 ± 2.15	6.81 ± 1.91	<.001	<.001
Lateral‐e', cm/s	12.32 ± 2.97	10.91 ± 3.02	10.11 ± 2.80	9.18 ± 2.58	<.001	<.001
Septal‐E/e' ratio	9.05 ± 2.38	9.92 ± 2.81	10.58 ± 3.09	11.22 ± 3.31	<.001	<.001
Lateral‐E/e' ratio	7.00 ± 1.89	7.59 ± 2.39	7.95 ± 2.45	8.41 ± 2.77	<.001	<.001
Average‐ E/e' ratio	8.03 ± 1.96	8.76 ± 2.40	9.27 ± 2.54	9.82 ± 2.75	<.001	<.001
Left atrial volume index, mL/m^2^	23.31 ± 6.72	24.58 ± 7.69	25.22 ± 7.65	25.08 ± 7.49	<.001	<.001
Global longitudinal strain, %	21.18 ± 2.44	20.44 ± 3.67	19.86 ± 3.71	18.89 ± 3.73	<.001	<.001
Global longitudinal strain <16%, *n* (%)	12 (2.60%)	74 (7.4%)	149 (10.1%)	453 (17.6%)	<.001	<.001
Left ventricular diastolic dysfunction, *n* (%)	78 (17.1%)	343 (34.2%)	646 (43.7%)	1482 (57.7%)	<.001	<.001

*Note*: Data are shown as median (Q1–Q3) for continuous variables and number(percentage) for categorical variables.

### 3.3 Association between MetS and LVDD and impaired GLS

The association between MetS and LVDD was detailed in Table 3 and Table S2. MetS participants had significantly higher risk of LVDD (OR: 1.40, 95% CI: 1.20–1.64) than non‐MetS participants after adjusted for clinical confounding factors (*p* < .05). Using the four levels of MetS elements groups, the multivariable analysis demonstrated increasing risk of LVDD with more MetS elements (*p* for trend <.001), compared with the 0 MetS elements, the OR of LVDD for 2 MetS elements was 1.41 (95% CI, 1.03–1.93), for ≥3 MetS elements was 1.88 (95% CI, 1.36–2.59). Table 4 and Table S2 showed the OR (95% CI) between the MetS and impaired GLS among individuals. Compared to the non‐MetS, the OR of impaired GLS for MetS was 1.26 (95% CI, 1.03–1.54). Compared with the 0 MetS elements, the OR of impaired GLS for ≥3 MetS elements was 2.05 (95% CI, 1.10–3.81). Similar results were found when restricting our analysis to those without medical treatment (Table S3). In sensitivity analysis, 1754 (31.9%) had MetS. Despite strict diagnostic criteria, MetS was also independently associated with LVDD and impaired GLS and the results were robust (Table S4). We also found compared with non‐MetS, MetS with all controlled targets had higher risk of LVDD, but not for impaired GLS; MetS with uncontrolled targets had higher risk of both LVDD and impaired GLS. (Table S5).

**TABLE 3:** Multivariable logistic regression between left ventricular diastolic dysfunction and metabolic syndrome.

Variables	Model 1		Model 2	
	OR (95% CI)	*p* value	OR (95% CI)	*p* value
Nonmetabolic syndrome	Ref		Ref	
Metabolic syndrome	2.30 (2.03–2.59)	<.001	1.40 (1.20–1.64)	<.001

Risk factor number = 0	Ref		Ref	
Risk factor number = 1	1.94 (1.44–2.62)	<.001	1.27 (0.92–1.75)	.140
Risk factor number = 2	2.70 (2.03–3.60)	<.001	1.41 (1.03–1.93)	.030
Risk factor number ≥3	4.90 (3.72–6.47)	<.001	1.88 (1.36–2.59)	<.001
*p* for trend	<.001		<.001	

*Note*: Model 1 adjusted age, sex. Model 2 adjusted age, sex, high‐school graduated or above, annual income ≥50 000 RMB, current smoking, systolic blood pressure, diastolic blood pressure, triglyceride, total cholesterol, high‐density lipoprotein cholesterol, low‐density lipoprotein cholesterol, fasting blood glucose, obesity, hypertension, type 2 diabetes mellitus, anti‐hypertensive drugs, hypoglycemic drugs, lipid‐lowering therapy, and left ventricular hypertrophy.

Abbreviations: CI, confidence interval; OR, odds ratio.

**TABLE 4:** Multivariable logistic regression between impaired global longitudinal strain and metabolic syndrome.

Variables	Model 1		Model 2	
	OR (95% CI)	*p* value	OR (95% CI)	*p* value
Nonmetabolic syndrome	Ref		Ref	
Metabolic syndrome	2.34 (1.98–2.77)	<.001	1.26 (1.03–1.54)	.023

Risk factor number = 0	Ref		Ref	
Risk factor number = 1	2.65 (1.42–4.95)	.002	1.65 (0.88–3.12)	.119
Risk factor number = 2	3.62 (1.98–6.60)	<.001	1.66 (0.89–3.09)	.107
Risk factor number ≥3	6.80 (3.79–12.22)	<.001	2.05 (1.10–3.81)	.023
*p* for trend	<.001		.053	

*Note*: Model 1 adjusted age, sex. Model 2 adjusted age, sex, current smoking, current drinking, systolic blood pressure, diastolic blood pressure, heart rate, triglyceride, total cholesterol, high‐density lipoprotein cholesterol, low‐density lipoprotein cholesterol, fasting blood glucose, obesity, hypertension, type 2 diabetes mellitus, anti‐hypertensive drugs, hypoglycemic drugs, and left ventricular hypertrophy.

Abbreviations: CI, confidence interval; OR, odds ratio.

### 3.4 Subgroup analysis

The results (Figure S1A) showed that a higher proportion of LVDD was observed in women with MetS, elderly with MetS, and obese people with MetS. Women with MetS had the greatest risk for LVDD (OR: 1.56, 95% CI: 1.24–1.96) (Table 5). We found a significant synergistic additive interaction between women and MetS, with RERI, AP, and S being 0.76 (0.02–1.50), 0.37 (0.20–0.53), and 3.15 (2.19–5.63), respectively (*p* < .05). However, the multiplicative interaction was not statistically significant (0.85, 95% CI: 0.66–1.10, *p* > .05). Elderly participants (age ≥ 60 years) had significantly higher risk of LVDD, especially elderly individuals with MetS (OR: 5.51, 95% CI: 4.48–6.76, *p* < .05). A significant synergistic additive interaction was found between elderly and MetS, with RERI, AP, and S being 35.65 (17.51–53.79), 0.86 (0.82–0.90), and 8.84 (6.54–11.97), respectively (*p* < .05). Obesity increased the risk of LVDD in MetS individuals (OR: 2.29, 95% CI: 1.82–2.89) but not non‐MetS individuals. A significant synergistic additive interaction between obesity and MetS, with RERI, AP, and S being 2.14 (95% CI: 0.66–3.62), 0.58 (0.47–0.69), and 4.99 (3.14–7.92), respectively (*p* < .05). However, the multiplicative interaction was not statistically significant (1.43, 95% CI: 0.95–2.13, *p* > .05).

**TABLE 5:** Subgroup analysis of the association between left ventricular diastolic dysfunction and metabolic syndrome.

Variables	MetS	Case/Participants	LVDD	Interaction on multiplicative scale	Interaction on additive scale		
			OR (95% CI)	OR (95% CI)	RERI (95% CI)	AP (95% CI)	S (95% CI)
Sex				Sex * MetS			
Men	No	425/1106	Ref	0.85 (0.66–1.10)	0.76 (0.02–1.50)	0.37 (0.20–0.53)	3.51 (2.19–5.63)
Men	Yes	562/1127	1.14 (0.91–1.41)				
Women	No	642/1829	1.17 (0.94–1.45)				
Women	Yes	920/1441	1.56 (1.24–1.96)				
Age (years)				Age * MetS			
<60	No	400/1842		0.74 (0.57–0.94)	35.65 (17.51–53.79)	0.86 (0.82–0.90)	8.84 (6.54–11.97)
<60	Yes	584/1364	1.47 (1.21–1.79)				
≥60	No	667/1093	5.06 (4.24–6.05)				
≥60	Yes	898/1204	5.51 (4.48–6.76)				
Obesity				Obesity * MetS			
No	No	983/2742	Ref	1.43 (0.95–2.13)	2.14 (0.66–3.62)	0.58 (0.47–0.69)	4.99 (3.14–7.92)
No	Yes	987/1818	1.19 (1.01–1.41)				
Yes	No	84/193	1.34 (0.94–1.90)				
Yes	Yes	495/750	2.29 (1.82–2.89)				

*Note*: Adjusted age, sex, current smoking, current drinking, systolic blood pressure, diastolic blood pressure, triglyceride, total cholesterol, high‐density lipoprotein cholesterol, low‐density lipoprotein cholesterol, fasting blood glucose, obesity, hypertension, type 2 diabetes mellitus, anti‐hypertensive drugs, hypoglycemic drugs, and left ventricular hypertrophy. Nonmetabolic syndrome was the reference group across the analysis.

Abbreviations: AP, the attributable proportion due to interaction; CI, confidence interval; LVDD, left ventricular diastolic dysfunction; MetS, metabolic syndrome; OR, odds ratio; RERI, the relative excess risk due to interaction; S, the synergy index.

The results (Figure S1B) showed that a high proportion of impaired GLS was observed in men with MetS, elderly with MetS, and obese people with MetS. Men had higher risk for impaired GLS than women, no matter whether with MetS (OR: 1.83, 95% CI: 1.30–2.58) or not (OR: 1.84, 95% CI: 1.33–2.53) (Table 6). The additive interaction indicator RERI was not statistically significant (*p* < .05). Elderly participants (age ≥ 60 years) had significantly higher risk of impaired GLS, no matter whether with MetS (OR: 1.68, 95% CI: 1.26–2.24) or not (OR: 1.39, 95% CI: 1.04–1.85, *p* < .05). The multiplicative interaction and RERI were not significant (*p* < .05). Obesity significantly increased the risk of impaired GLS in MetS individuals (OR: 2.88, 95% CI: 2.21–3.76, *p* < .05). A significant synergistic additive interaction was found between obesity and MetS, with RERI, AP, and S being 3.37 (95% CI: 0.50–6.24), 0.66 (0.55–0.77), and 5.74 (3.44–9.56), respectively (*p* < .05). However, the multiplicative interaction was not statistically significant (1.63, 95% CI: 0.99–2.69, *p* < .05).

**TABLE 6:** Subgroup analysis of the association between impaired global longitudinal strain and metabolic syndrome.

Variables	MetS	Case/Participants	Impaired GLS	Interaction on multiplicative scale	Interaction on additive scale		
			OR (95% CI)	OR (95% CI)	RERI (95% CI)	AP (95% CI)	S (95% CI)
Sex				Sex * MetS			
Women	No	93/1829	Ref	0.68 (0.48–0.97)	2.61 (−0.62–5.86)	0.53 (0.31–0.75)	3.01 (1.97–4.61)
Women	Yes	211/1441	1.45 (1.07–1.96)				
Men	No	142/1106	1.84 (1.33–2.53)				
Men	Yes	242/1127	1.83 (1.30–2.58)				
Age (years)				Age* MetS			
<60	No	127/1842	Ref	1.05 (0.73–1.48)	1.15 (−0.18–2.49)	0.42 (0.22–0.62)	3.12 (2.18–4.45)
<60	Yes	233/1364	1.15 (0.87–1.52)				
≥60	No	108/1093	1.39 (1.04–1.85)				
≥60	Yes	220/1204	1.68 (1.26–2.24)				
Obesity				Obesity * MetS			
No	No	209/2742	Ref	1.63 (0.99–2.69)	3.37 (0.50–6.24)	0.66 (0.55–0.77)	5.74 (3.44–9.56)
No	Yes	235/1818	1.07 (0.84–1.36)				
Yes	No	26/193	1.63 (1.03–2.56)				
Yes	Yes	218/750	2.88 (2.21–3.76)				

*Note*: Adjusted age, sex, current smoking, current drinking, systolic blood pressure, diastolic blood pressure, heart rate, triglyceride, total cholesterol, high‐density lipoprotein cholesterol, low‐density lipoprotein cholesterol, fasting blood glucose, obesity, hypertension, type 2 diabetes mellitus, anti‐hypertensive drugs, hypoglycemic drugs, and left ventricular hypertrophy. Nonmetabolic syndrome was the reference group across the analysis.

Abbreviations: AP, the attributable proportion due to interaction; CI, confidence interval; GLS, global longitudinal strain; MetS, metabolic syndrome; OR, odds ratio; RERI, the relative excess risk due to interaction; S, the synergy index.

## 4 DISCUSSION

This study has comprehensively demonstrated the association between MetS and LVDD or LVSD. MetS was independently associated with high risk of LVDD and LVSD after adjusting for hypertension, obesity, and diabetes. Being female, elderly, obese, and having MetS had a significant synergistic additive interaction regarding the risk of LVDD. Obesity and MetS had a significant synergistic additive interaction regarding the risk of impaired GLS. These findings may help to identify a high‐risk population of subclinical LVD and to reduce related cardiovascular disease.

Our findings suggest that MetS individuals had higher prevalence of LVDD and impaired GLS. The results of this study support the findings in other population that MetS was associated with worse diastolic function^22^ and worse LV longitudinal strain.^3^, ^4^, ^8^, ^23^, ^24^ In Hispanics/Latinos, MetS was independently associated only with decreased longitudinal strain in apical two‐ and four‐chamber views.^3^ In another study, MetS was not independently associated with impaired GLS with cutoff values 16.1%, 14.8%, or 13.5%^4^ after adjusted confounders. However, we found MetS was independently associated with impaired GLS with cutoff value 16% even after being adjusted for hypertension, obesity, and diabetes. Our study confirmed the controversy using a large population, comprehensively evaluated LVDD with e' velocity, E/e' ratio, LAVI, and E/A ratio, not as previously one or two indicators, and accurately assessed subclinical systolic function with impaired GLS in three chambers, not as previously one or two chambers. We further demonstrated as metabolic risk elements increased, the risk of LVDD and LVSD increased.

The main clinical implications of this study relate to public health. A recent study^5^ has shown a higher prevalence of MetS among women, the elderly, and urban residents. Although the association of MetS and LVD has been investigated, identifying which subgroups had high risk of LVD and related cardiovascular disease is vital for guiding intervention when medical resources are limited. Recent study^25^ from Korea recruited employees aged 18 years or elderly found MetS had an independent association with LVDD in men, not women. Among middle‐aged and elderly Korean patients at high risk of cardiovascular disease,^26^ the impact of MetS on LVDD was more pronounced in women than in men. Another study that included middle‐aged and elderly participants found consistent results.^22^ We also found the significant synergistic additive interaction regarding the risk of LVDD between women and MetS. The reason for the controversy may be the increased proportion of postmenopausal women^27^ as menopause generally occurs around the age of 50. The loss of hormones protection may lead to the sharp increase in hypertension and dyslipidemia.^28^ This confirmed the vital role of sex hormonal effects in the development of LVDD in relation to MetS. MetS and hormonal derange are strongly associated with increased LV filling pressure, cardiac fibrosis, and an increased risk of heart failure with preserved ejection fraction (HFpEF). Accumulating evidence points to an enhanced risk of HFpEF in women with obesity, diabetes, and MetS^29^ than men. We further found the impact of MetS on impaired GLS was more pronounced in men than women by significant multiplicative interaction. Previous study showed men had lower GLS than women.^30^ Few studies examined the impact of sex and MetS interaction on impaired GLS.

Obesity combined with MetS had the lowest e' and GLS^31^ and highest E/e' ratio.^8^ Previous study usually compared the difference between the groups and explore the relationship with linear regression. We first explored the multiplicative and additive interaction between obesity and MetS and found a significant synergistic interaction regarding the risk of LVDD and LVSD. Obesity provided an important link between metabolic disorders and systemic inflammation, and thus, is a major determinant of HFpEF.^32^ The systemic inflammatory response would be amplified by visceral adiposity amplifies. Obesity and elevated waist circumference had a synergistic interaction regarding the risk of HFpEF. Elderly participants were associated with increased risk of LVDD, as demonstrated by a previous study.^7^ The prevalence of MetS in the total population peaked around the age of 60 years.^33^ The participants with mean age 56.59 years in the current study had higher prevalence of MetS. Whether the multiplicative and additive interaction between elderly and MetS regarding LVD was unknown. We suggested the significant multiplicative and additive interaction between elderly and MetS regarding LVDD, not impaired GLS.

### 4.1 Strengths and limitations

The current study has some strengths. The sample size of this study regarding evaluation of LVDD and impaired GLS among MetS individuals was large. The project was sponsored by the Chinese government and the data quality control is strictly unified. The association between MetS and LVD was demonstrated from several aspects and most important by first evaluating the additive interaction in order to identify high‐risk populations. There may be some possible limitations in this study. First, we investigated the MetS elements related to LVDD and impaired GLS; however, we cannot explain causality due to the cross‐sectional study. Second, the participants were recruited in southern China and were not nationally representative. Third, other clinical risk factors associated with LVD were not included in our study, such as inflammation indicators, serum creatinine, and uric acid. Fourth, although we used the FBG values obtained from fingertip blood samples for the definitions of MetS, the sensitivity analysis results were robust. Fifth, there are too many observations from which a distinct conclusion cannot be achieved and further investigation is needed to explore the causality.

## 5 CONCLUSIONS

MetS was independently associated with LVDD and impaired GLS. Participants with MetS might represent an at‐risk population to target interventions for primordial prevention of LVDD and LVSD. From the public health implications, prevention of MetS in women, elderly, and obese individuals might result in a greater reduction of LVDD and LVSD in a cardiovascular high‐risk population.

## AUTHOR CONTRIBUTIONS

Methodology: Dan Zhou, Zhiqiang Nie, Chaolei Chen. Formal analysis: Dan Zhou, Zhiqiang Nie. Resources: Dan Zhou, Zhongwen Ye, Songyuan Luo, Mengqi Yan, Jiabin Wang. Data curation: Dan Zhou, Zhiqiang Nie. Writing—original draft preparation: Dan Zhou. Writing—review and editing: Dan Zhou, Yingqing Feng. Visualization: Dan Zhou, Yingqing Feng. Supervision: Dan Zhou, Zhiqiang Nie, Chaolei Chen, Songyuan Luo, Mengqi Yan, Jiabin Wang, Yingqing Feng. Funding acquisition: Yingqing Feng. All authors read and approved the final manuscript.

## FUNDING INFORMATION

This work was supported by the Ministry of Finance of China and National Health and Family Planning Commission of China, the Key Area R&D Program of Guangdong Province (No. 2019B020227005), the Climbing Plan of Guangdong Provincial People's Hospital (DFJH2020022), and Guangdong Provincial Clinical Research Center for Cardiovascular disease (2020B1111170011), Guangdong Provincial Key Laboratory of Coronary Heart Disease Prevention (2017B030314041).

## DISCLOSURE

The authors declare no conflict of interest.

## Supporting information


**DATA S1:** Supporting Information.

## Data Availability

The data sets generated and/or analyzed during the current study are not publicly available due to shared copyright of sensitive data but are available from the corresponding author on reasonable request.
