# Supplementary material for: Positive additive interaction effects of age, sex, obesity, and metabolic syndrome on left ventricular dysfunction
Source: J Diabetes. 2023 Sep 24;16(1):e13478. doi: 10.1111/1753-0407.13478 (PMC10809287; doi:10.1111/1753-0407.13478)

Table S1 Collinearity test and Variance Inflation Factor (VIF) value

| Variable | VIF |
| --- | --- |
| age | 1.26 |
| sex | 1.87 |
| current smoking | 1.61 |
| current drinking | 1.12 |
| systolic blood pressure | 3.16 |
| diastolic blood pressure | 2.16 |
| heart rate | 1.07 |
| triglyceride | 1.65 |
| total cholesterol | 6.86 |
| high-density lipoprotein cholesterol | 2.23 |
| low-density lipoprotein cholesterol | 5.17 |
| fasting blood glucose | 1.89 |
| obesity | 1.12 |
| hypertension | 2.99 |
| Type 2 diabetes mellitus | 2.34 |
| anti-hypertensive drugs | 1.39 |
| hypoglycemic drugs | 1.54 |
| left ventricular hypertrophy. | 1.11 |
| Metabolic Syndrome | 1.70 |

Table S2 Univariable logistic regression of MetS and LVDD, impaired GLS.

|  | LVDD |  | Impaired GLS |  |
| --- | --- | --- | --- | --- |
| Variable | OR (95%CI) | P value | OR (95%CI) | P value |
| Non-MetS | Ref |  | Ref |  |
| MetS | 2.39(2.14-2.66) | <0.001 | 2.46(2.08-2.91) | <0.001 |

OR: odds ratio; CI: confidence interval

Table S3 Subgroup analysis in participants without anti-hypertensive drugs, hypoglycemic drugs or lipid-lowering therapy.

|  | LVDD |  | GLS<16% |  |
| --- | --- | --- | --- | --- |
|  | OR (95%CI) | P value | OR (95%CI) | P value |
| Case/Participants | 1445/3748 |  | 414/3748 |  |
| Non-metabolic syndrome | Ref |  | Ref |  |
| Metabolic syndrome | 1.34(1.12-1.60) | 0.002 | 1.34(1.04-1.71) | 0.022 |
| Risk factor number =0 | Ref |  | Ref |  |
| Risk factor number =1 | 1.22(0.88-1.69) | 0.238 | 1.83(0.96-3.48) | 0.066 |
| Risk factor number =2 | 1.36(0.98-1.87) | 0.066 | 1.93(1.02-3.63) | 0.042 |
| Risk factor number≥3 | 1.71(1.22-2.40) | 0.002 | 2.45(1.29-4.64) | 0.006 |
| P for trend | 0.004 |  | 0.027 |  |

Adjusted age, sex, current smoking, current drinking, systolic blood pressure, diastolic blood pressure, heart rate, triglyceride, total cholesterol, high-density lipoprotein cholesterol, low-density lipoprotein cholesterol, fasting blood glucose, obesity, hypertension, Type 2 diabetes mellitus, anti-hypertensive drugs, hypoglycemic drugs, left ventricular hypertrophy. GLS: Global longitudinal strain, LVDD: left ventricular diastolic dysfunction.

OR: odds ratio; CI: confidence interval.

Table S4 Multivariable logistic regression between LVDD, impaired GLS and metabolic syndrome in sensitivity analysis.

|  | LVDD |  | Impaired GLS |  |
| --- | --- | --- | --- | --- |
|  | OR (95%CI) | P value | OR (95%CI) | P value |
| Non-metabolic syndrome | Ref |  | Ref |  |
| Metabolic syndrome | 1.29(1.10-1.52) | 0.002 | 1.38(1.13-1.68) | <0.001 |
| Risk factor number =0 | Ref |  | Ref |  |
| Risk factor number =1 | 1.31(1.02-1.68) | 0.03 | 2.09(1.24-3.51) | <0.005 |
| Risk factor number =2 | 1.64(1.27-2.12) | <0.001 | 2.46(1.47-4.12) | <0.001 |
| Risk factor number≥3 | 1.93(1.46-2.56) | <0.001 | 3.48(2.09-5.79) | <0.001 |
| P for trend | <0.001 |  | <0.001 |  |

Adjusted age, sex, current smoking, current drinking, systolic blood pressure, diastolic blood pressure, heart rate, triglyceride, total cholesterol, high-density lipoprotein cholesterol, low-density lipoprotein cholesterol, fasting blood glucose, obesity, hypertension, Type 2 diabetes mellitus, anti-hypertensive drugs, hypoglycemic drugs, left ventricular hypertrophy. GLS: Global longitudinal strain, LVDD: left ventricular diastolic dysfunction. OR: odds ratio; CI: confidence interval.

**In sensitivity analysis, elevated FBG in metabolic syndrome definition which only defined by using hypoglycemic drugs in the following table. Finally, 1754(31.9%) was defined metabolic syndrome.**

**Table S5** Multivariable logistic regression between LVDD, impaired GLS and metabolic syndrome.

|  | LVDD |  | Impaired GLS |  |
| --- | --- | --- | --- | --- |
|  | OR (95%CI) | P value | OR (95%CI) | P value |
| Non-MetS | Ref |  | Ref |  |
| MetS with three controlled targets | 1.63(1.07-2.47) | 0.022 | 1.35(0.79-2.30) | 0.262 |
| MetS with uncontrolled targets | 1.28(1.10-1.48) | 0.001 | 1.25(1.02-1.53) | 0.028 |

Adjusted age, sex, high-school graduated or above, annual income ≥50000 RMB, current smoking, systolic blood pressure, diastolic blood pressure, triglyceride, total cholesterol, high-density lipoprotein cholesterol, low-density lipoprotein cholesterol, fasting blood glucose, obesity, hypertension, Type 2 diabetes mellitus, anti-hypertensive drugs, hypoglycemic drugs, lipid-lowering therapy, left ventricular hypertrophy.

OR: odds ratio; CI: confidence interval.

Figure S1A The prevalence of LVDD in different subgroup.


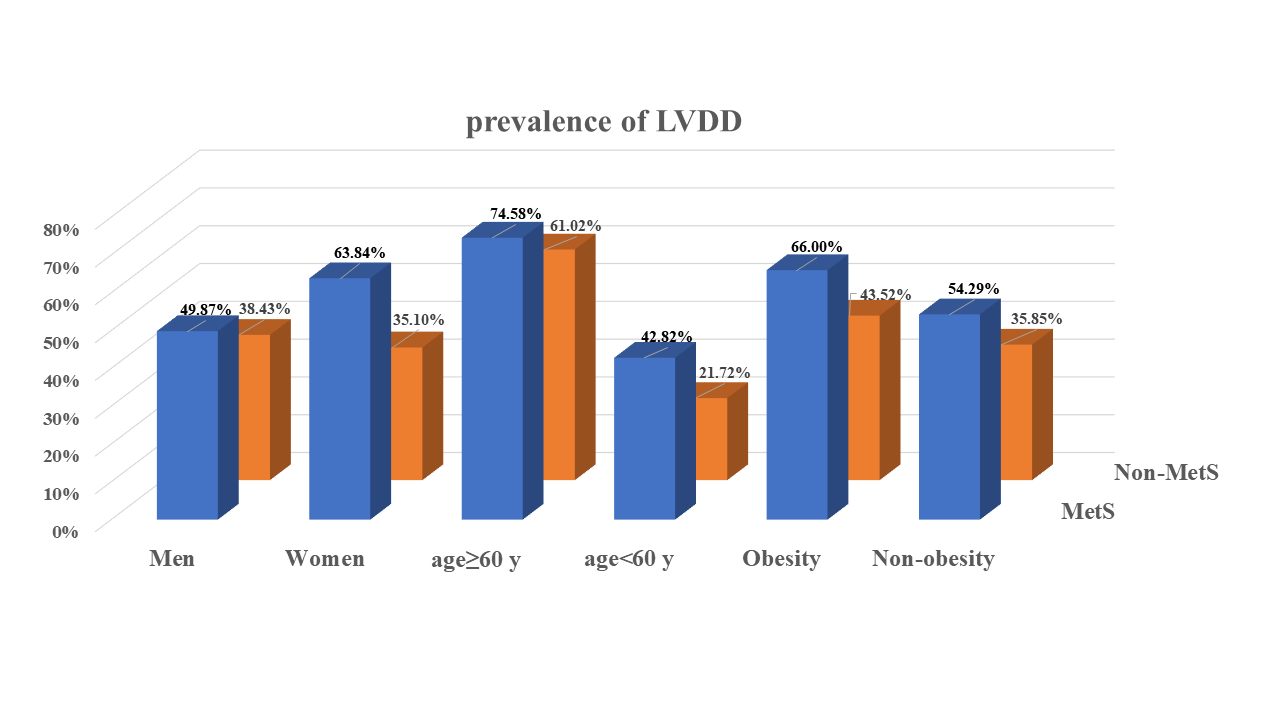


Figure S1B The prevalence of GLS in different subgroup.


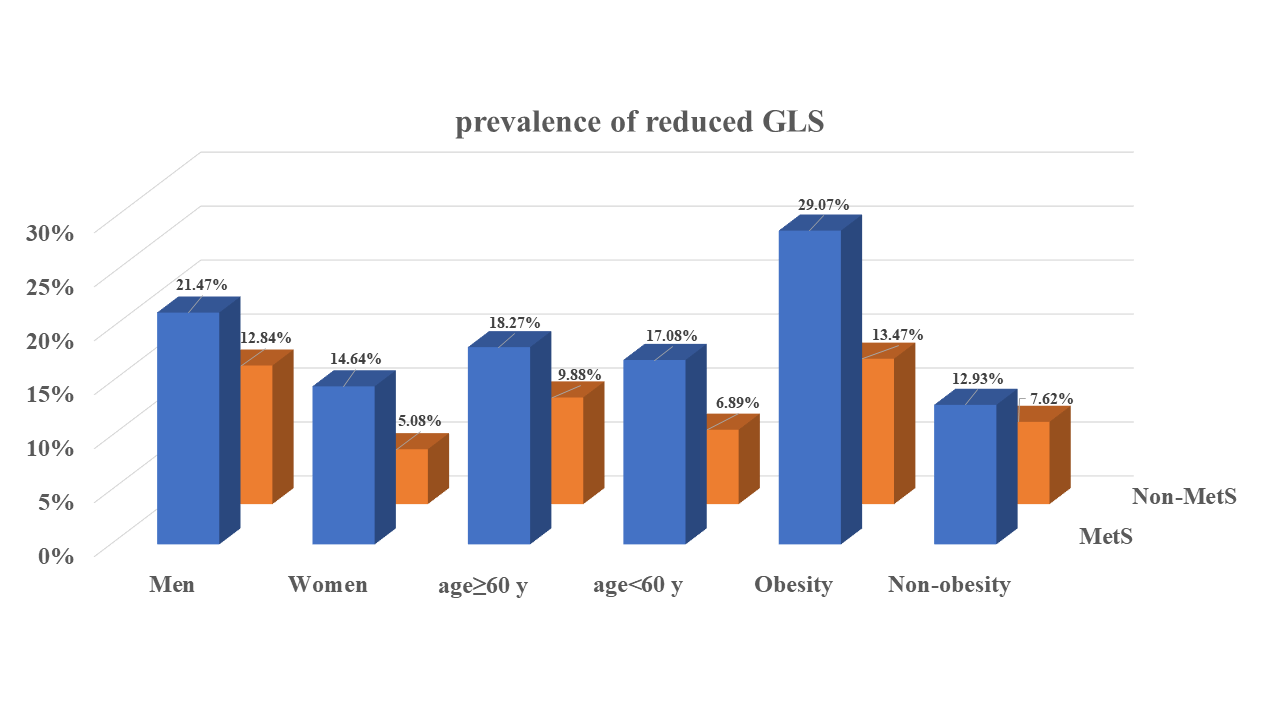

Supplement: Supplementary file 1 — DATA S1: Supporting Information. [file JDB-16-e13478-s001.docx]
